# Synthesis, Characterization, and Evaluation of a Novel Amphiphilic Polymer RGD-PEG-Chol for Target Drug Delivery System

**DOI:** 10.1155/2014/546176

**Published:** 2014-01-21

**Authors:** Shi Zeng, Fengbo Wu, Bo Li, Xiangrong Song, Yu Zheng, Gu He, Cheng Peng, Wei Huang

**Affiliations:** ^1^State Key Laboratory of Biotherapy and Department of Pharmacy, West China Hospital, Sichuan University, No. 37 Guoxue Alley, Chengdu, Sichuan 610041, China; ^2^State Key Laboratory Breeding Base of Systematic research, Development and Utilization of Chinese Medicine, Chengdu University of Traditional Chinese Medicine, Chengdu 611137, China

## Abstract

An amphiphilic polymer RGD-PEG-Chol which can be produced in large scale at a very low cost has been synthesized successfully. The synthesized intermediates and final products were characterized and confirmed by ^1^H nuclear magnetic resonance spectrum (^1^H NMR) and Fourier transform infrared spectrum (FT-IR). The paclitaxel- (PTX-) loaded liposomes based on RGD-PEG-Chol were then prepared by film formation method. The liposomes had a size within 100 nm and significantly enhanced the cytotoxicity of paclitaxel to B16F10 cell as demonstrated by MTT test (IC_50_ = 0.079 **μ**g/mL of RGD-modified PTX-loaded liposomes compared to 9.57 **μ**g/mL of free PTX). Flow cytometry analysis revealed that the cellular uptake of coumarin encapsulated in the RGD-PEG-Chol modified liposome was increased for HUVEC cells. This work provides a reasonable, facile, and economic approach to prepare peptide-modified liposome materials with controllable performances and the obtained linear RGD-modified PTX-loaded liposomes might be attractive as a drug delivery system.

## 1. Introduction

During the last decade, many reports have been focused on developing colloidal carrier systems for a variety of anticancer drugs to achieve targeted drug delivery to tumor sites and improve their intracellular uptake. Amphiphilic polymers have received increasing attention because of their desired chemical, interfacial, mechanical, and biological functions, which make them a suitable platform for sustained drug release [1–4] and a promising carrier for therapeutic agents (especially poorly soluble drugs) [5]. Meanwhile, the development of improved synthetic methodologies has greatly supported the growth of this field [6, 7].

Liposomal delivery has been extensively investigated in cancer treatment. To avoid rapid clearance of liposomes from the circulation by the reticuloendothelial system, sterically stabilized liposomes (SSL) have been developed. The moieties of poly(ethylene glycol)s (PEGs) as membrane active agents are commonly used within modern pharmaceutical chemistry, because it could extend in “mushroom” or “brush” chain conformation from the membrane plane towards the water solution and form thick hydrophilic layers at the membrane surface [8]. The functionalization of PEG allows subsequent attachment of biologically active or even cell-targeting molecules to prepare sterically stabilized liposomes which retain long survival time in circulation and target recognition [9]. In our work, PEG2000 was chosen since it can covalently attach to the lipid head group, undergo steric exclusion from the liposome surface, and play an important role in thermodynamic stability of liposomes by dehydrating the lipid bilayer [10–14].

Although differential accumulation of liposomes carrying drugs in tumor sites relative to normal tissues can be achieved by the effect of enhanced permeability and retention (EPR), it is still necessary to improve inefficient intracellular uptake of the entrapped anticancer drugs by the tumor cells by active targeting strategy [15–17]. Integrin is a family of cell surface receptors responsible for anchoring cells to the extracellular matrix (ECM) and they are overexpressed in the melanoma tumor cells and tumor endothelial cells [18]. The RGD (Arg-Gly-Asp) sequence can bind to several integrins such as integrin *a*_v_ *β*_3_ and *a*_5_ *β*_1_ [19]. In this paper, we used a linear RGD peptide to construct an amphiphilic polymer cholesterol-poly(ethylene glycol)-RGD and used it to establish a RGD-modified liposomal drug delivery system for paclitaxel (PTX), a natural antimicrotubule agent for treating a variety of solid tumors, such as ovarian cancer, breast cancer, and lung cancer [20, 21].

RGD-modified polymers have been investigated extensively and there are also reports about Lipid-PEG-RGD polymers which are similar to our work [22, 23]. These works are very extraordinary, impressive and laid a solid foundation for our study. Compared with their outstanding work, however, we also have our own advantages. In our work, we choose cholesterol as biomembrane anchor instead of artificial lipids such as 1,2-distearoyl-sn-glycero-3-phosphoethanolamine (DSPE) because of the fact that cholesterol gives liposomal systems similar mechanical and biological functions compared to DSPE [24–27] and that the cost of cholesterol is over 100 times lower than that of DSPE, which means it can be easily synthesized in large amounts on an industrial scale. In our work, we choose ordinary PEG instead of Fmoc-PEG-CO_2_NHS, which had been reported, since ordinary PEG is far more cost-efficient. Also the synthesis route we designed is more simple and facile, and there is no need to use purification methods such as HPLC which cost more money and time. In this way, our method leads to higher product rate and the possibility of producing in large scale.

The aim of this paper was to synthesize cholesterol-poly(ethylene glycol)-RGD amphiphilic polymer (RGD-PEG-Chol) and develop an RGD-modified PTX-loaded liposome for targeted drug delivery. The RGD-modified PTX-loaded liposome has been extended to evaluate physicochemical properties such as particle size, morphologies, and zeta potential. The drug release profile, cellular uptake, and antitumor activity of PTX liposomes were also investigated* in vitro.*

## 2. Experimental Section 

### 2.1. Materials

Cholesterol (Chol) was obtained from BoAo Biological Technology (Shanghai, China). Monomethoxy poly(ethylene glycol) (MW 2000, mPEG2000), poly(ethylene glycol) (MW 2000, PEG2000), 3-[4,5-dimethylthiazol-2-yl]-2,5-diphenyltetrazolium bromide (MTT), 1-(3-dimethylaminopropyl)-3-ethylcarbodiimide hydrochloride (EDCI), and 4-dimethylaminopyridine (DMAP) were purchased from Sigma-Aldrich (USA). 1,8-Diazabicyclo [5.4.0] undec-7-ene (DBU), coumarin-6, fmoc-L-phenylalanine (fmoc-phe), and succinic anhydride (suc) were provided by Energy Chemical (Shanghai, China). 3-Maleimidopropionic acid N-succinimidyl ester (BMPS) was purchased from Chengdu Boke Co. Ltd. (Chengdu, China). Soya phosphatidylcholine (SPC) was purchased from Lucas Meyer (Hamburg, Germany). Paclitaxel (PTX) was purchased from Yihe Biological Technology (Shanghai, China). Cysteine-glycine-arginine-glycine-asparagine-serine (CGRGDS) hexapeptide was obtained from Chengdu Kaijie Co. Ltd. (Chengdu, China). All other solvents and reagents were of chemical grade and used without other purification. Ultrapure water from Milli-Q water system was used to prepare the aqueous solutions.

### 2.2. Synthesis of Methoxy Poly(ethylene glycol)-Cholesterol Conjugate

**Scheme 1:**
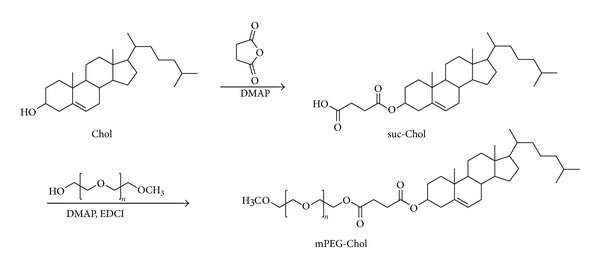
Synthetic route of mPEG-Chol polymer.

Methoxy poly(ethylene glycol)-cholesterol conjugate (mPEG-Chol) was synthesized according to literature [28–30] with minor modifications. As is displayed in Scheme 1, firstly, a solution of dichloromethane (100 mL) containing cholesterol (5.0 g, 12.9 mmol), DMAP (1.6 g, 12.9 mmol), and succinic anhydride (6.5 g, 66.7 mmol) was stirred at room temperature for 48 h. Thereafter most dichloromethane was removed using rotary evaporator and the crude solution was poured into acetic acid (150 mL). The precipitates were filtered and washed three times with acetic acid, and cholesterol monosuccinate (Chol-suc) (5.8 g 92.5%) was finally acquired by vacuum drying overnight. Secondly, MPEG (2.0 g, 1.0 mmol), Chol-suc (0.72 g, 1.5 mmol), DMAP (0.13 g, 1.0 mmol), and EDCI (0.48 g, 2.5 mmol) were dissolved in dichloromethane (100 mL) and refluxed at room temperature for 24 h. The reaction mixture was then washed with 1N HCl solution, saturated in NaCl solution, and dried over anhydrous Na_2_SO_4_. The solvent was evaporated and the residue was purified with column chromatography (methanol : dichloromethane = 1 : 20) to give mPEG-Chol (2.12 g, 86.1%).

### 2.3. Synthesis of Cholesterol-Poly(ethylene glycol)-RGD Peptides Conjugate

**Scheme 2:**
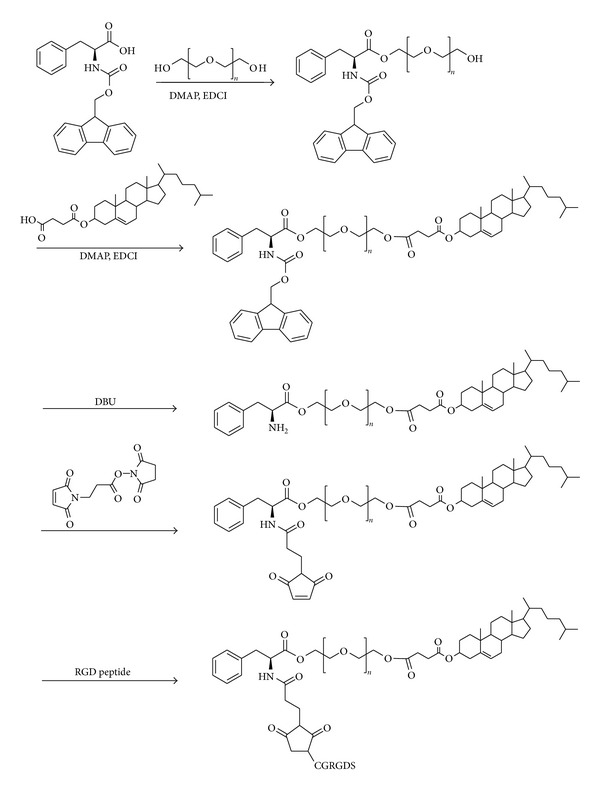
Synthetic route of RGD-PEG-Chol polymer.

The synthesis process of cholesterol-poly(ethylene glycol)-RGD conjugate (RGD-PEG-Chol) was shown in Scheme 2.

#### 2.3.1. Synthesis of Poly(ethylene glycol)-Phe-Fmoc Conjugate (PEG-Phe-Fmoc)

A dichloromethane solution (150 mL) of fmoc-phe (1.45 g, 3.75 mmol), DMAP (0.31 g, 2.5 mmol), EDCI (0.72 g, 3.75 mmol), and PEG (5.0 g, 2.5 mmol) was stirred at room temperature for 48 h. After completion of the reaction, the solution was washed with 1N HCl solution, saturated in NaCl solution, and then dried over anhydrous Na_2_SO_4_. The solvent was evaporated and the residue was purified with column chromatography (methanol : dichloromethane = 1 : 60) to give the monofunctional product, poly(ethylene glycol)-phe-fmoc conjugate (PEG-Phe-Fmoc) (5.2 g, 87.8%).

#### 2.3.2. Synthesis of Cholesterol-Poly(ethylene glycol)-Phe-NH_**2**_ Conjugate (Chol-PEG-Phe-NH_**2**_)

The mixture of PEG-Phe-Fmoc (5.0 g, 2.1 mmol), Chol-suc (1.2 g, 2.5 mmol), DMAP (0.25 g, 2.1 mmol), and EDCI (0.8 g, 4.2 mmol) in dichloromethane (150 mL) was stirred at room temperature for 24 h. After completion of the reaction, the solution was washed with 1N HCl solution, saturated NaCl solution, and dried over anhydrous Na_2_SO_4_. The solution was concentrated under vacuum and cholesterol-poly(ethylene glycol)-phe-fmoc conjugate (Chol-PEG-Phe-Fmoc) (5.2 g, 87.2%) was obtained after being purified on a silica-gel column chromatography (methanol : dichloromethane = 1 : 40). Chol-PEG-Phe-Fmoc (3.0 g, 1.1 mmol) dissolved in dichloromethane (50 mL) was refluxed at room temperature for 10 min; then DBU (0.19 g, 1.3 mmol) was added. After being refluxed at room temperature for 40 min, the solution was washed directly with 1N HCl solution, saturated in NaCl solution, and dried over anhydrous Na_2_SO_4_. Dichloromethane was removed using rotary evaporator and the crude solution was poured into ethyl ether (100 mL). The precipitated solid was separated by filtration, washed three times with ethyl ether, finally, the Chol-PEG-Phe-NH_2_ (2.4 g, 83.6%) was obtained by vacuum drying overnight.

#### 2.3.3. Synthesis of RGD-PEG-Chol

A mixture of Chol-PEG-Phe-NH_2_ (2.0 g, 0.76 mmol) and BMPS (0.24 g, 0.92 mmol) in DMSO (50 mL) was stirred at room temperature for 24 h to give a 3-maleimidopropionic acid linker on PEG chain. The peptide CGRGDS (0.55 g, 0.92 mmol) was added and stirred for another 24 h. After completion of the reaction, the solution was dialyzed successively against H_2_O (membrane tubing, molecular weight cutoff 1000 Da). The product after dialysis was then lyophilized and RGD-PEG-Chol (1.2 g, 47.1%) was obtained.

### 2.4. Cell Cultures

The murine B16F10 cell lines were obtained from the Basic Medical Cell Center, Chinese Academy of Medical Science (CAMS, Beijing, China) and were cultured in DMEM medium (Gibco, Paisley, UK) supplemented with 100 units/mL penicillin, 100 units/mL streptomycin, and 10% heat-inactive fetal calf serum and incubated in a humidified atmosphere containing 5% CO_2_ at 37°C. Human umbilical vein endothelial cells (HUVECs) were isolated from umbilical cords, cultured in EGM-2 medium supplemented with 100 units/mL penicillin and 100 units/mL streptomycin. The concentration of cells was determined by counting trypsinized cells with a hemocytometer.

### 2.5. Liposomes Preparation and Drug Loading

Conventional liposomes (CL) were prepared by a thin film dispersion method. Accurately weighed SPC (39 mg), Chol (13 mg), mPEG-Chol (10 mg), and PTX (0.667 mg) were first dissolved in 4 mL chloroform in a round-bottom flask, dried into transparent thin film on a rotary evaporator under vacuum at 30°C, and followed by vacuum dry overnight to remove any traces of organic solvent. The lipid film was hydrated by 8 mL of Milli-Q water at 60°C for 20 min, after which the suspension was dispersed by a probe-type ultrasonicator for 5 min and sequentially extruded through a 0.8 *μ*m and 0.2 *μ*m pore size polycarbonate filter (Nuclepore, USA).

RGD-modified liposomes (CGRGDS) were obtained by the same method above, except that mPEG-Chol and RGD-PEG-Chol polymers with different mass ratios (from 10 mg/0 mg to 0 mg/10 mg) were dissolved in chloroform along with SPC, Chol and PTX, or coumarin-6 before the thin film was prepared.

To determine the drug loading (DL) and encapsulation efficiency (EE) of the PTX-loaded liposomes, 1 mL liposome was diluted in 1 mL methanol, and then the PTX content was determined by high-performance liquid chromatography (HPLC). In the analysis, a LC-20AD (Shimadzu Corp., Japan) apparatus equipped with a SPD-20A UV detector (Shimadzu Corp., Japan) and a DIONEX Acclaim 120 RP-C18 column (DIONEX Corp., USA) (250 × 4.6 mm) were used. The mobile phase was composed of 60 : 40 (v/v) acetonitrile-water with a flow rate of 1 mL/min and the detection wavelength was set at 227 nm. The DL and EE of the liposomes were calculated using the following formulas:

(1) $$\begin{array}{c} \text{DL}=\frac{\;\text{weight}\;\;\text{of}\;\;\text{drug}\;\;\text{in}\;\;\text{liposome}}{\text{weight}\;\;\text{of}\;\;\text{feeding}\;\;\text{polymer}\;\;\text{and}\;\;\text{drug}}\times 100\%,\\ \text{EE}=\frac{\text{weight}\;\;\text{of}\;\;\text{drug}\;\;\text{in}\;\;\text{liposome}}{\text{weight}\;\;\text{of}\;\;\text{drug}\;\;\text{feed}}\times 100\%. \end{array}$$

### 2.6. Size Determination and Zeta Potential

Particle size of the liposome was determined by Dynamic light scattering (DLS) with a Zetasizer Nano ZS-90 instrument (Malvern Instruments, Malvern, UK). Zeta potential was monitored using the same instrument as size measurement. Zeta limits ranged from −150 to 150 mV. The liposome suspension was diluted 10 times before measurement. During the progress, refractive index was 1.5000, and temperature was kept at 25°C. Each test was measured for 3 times and mean value was taken.

### 2.7. Transmit Electronic Microscopy (TEM)

A transmission electron microscope (TEM) was used to observe the morphology of prepared liposome. Liposome was diluted 10 times with distilled water and placed on a copper grid covered with nitrocellulose. The sample was negatively stained with phosphotungstic acid and dried at room temperature, after which TEM images were taken by a transmission electron microscope (H-6009IV, Hitachi, Japan).

### 2.8. *In Vitro* Drug Release

The release profiles of PTX from RGD-modified liposome and free PTX were investigated by the dialysis method. 1 mL drug loaded liposome or free PTX was placed into dialysis bags (molecular weight cutoff = 3500) which were then incubated in 50 mL phosphate buffered solution (PBS) (PH 7.4 or PH 5.5, 0.01 M) containing Tween 80 (0.5% wt) at 37°C with gentle shaking (100 rpm). At given time points, 1 mL dialysis medium was withdrawn and replaced with same volume of fresh buffer. To calculate cumulative amount of released PTX, the samples were analyzed by HPLC. All the results were the mean value of three test runs and all data were shown as the mean ± SD.

### 2.9. Cellular Uptake of Liposomes by Flow Cytometry Analysis

An aliquot of 1.5 mL of HUVEC cells suspension (6 × 10^4^ cells/well) was seeded in a six-well tissue culture plate (Corning, NY, USA) and was incubated for 24 h at 37°C. Then 30 *μ*L coumarin-6 loaded conventional liposomes or RGD-modified liposomes were added to each well, respectively, and the final concentration of coumarin-6 is 40 ng/mL. The plates were incubated at 37°C for another 1 h, after which the medium was discarded and cell monolayer was suspended by treatment with trypsin and washed three times with cold PBS. Then the cell samples were examined by a flow cytometer (EPICS Elite ESP, Beckman Coulter, USA). The intracellular coumarin-6 was excited with an argon laser (488 nm), and fluorescence was detected at 525 nm. Files were collected from 10,000 gated events.

### 2.10. *In Vitro* Cytotoxicity Assay

A comparison of *in vitro* cytotoxicity of various PTX formulations was performed on B16F10 cells with an *in vitro* proliferation using MTT method. Briefly, 3000 cells in 100 *μ*l DMEM were plated in 96-well plates and incubated for 24 h at 37°C in humanized atmosphere containing 5% CO_2_. These cells were exposed to different concentrations of free PTX, blank liposome, PTX-loaded liposome (mPEG/PTX Lipo), and RGD-modified PTX-loaded liposome (RGD/PTX Lipo) at 37°C for 48 h. 20 *μ*l of MTT solution (5 mg/mL dissolved in physiological saline) was added and incubated for another 4 h, and then the media were replaced with 150 *μ*l DMSO. After slight shake for 10 min, the absorbance was read on a Spectramax M5 Microtiter Plate Luminometer (Molecular Devices, US) at dual wavelength of 490 nm. Each experiment was repeated three times in triplicate (*n* = 9). The data reports represented the means of triplicate measurement.

### 2.11. Statistics Analysis

All data were reported as means ± standard deviation (SD). Statistical significance between pairs of samples was measured using 2-tailed Student's *t*-test. A value of *P* < 0.05 was considered statistically significant.

## 3. Results and Discussion

### 3.1. Synthesis of RGD-PEG-Chol

**Figure 1:**
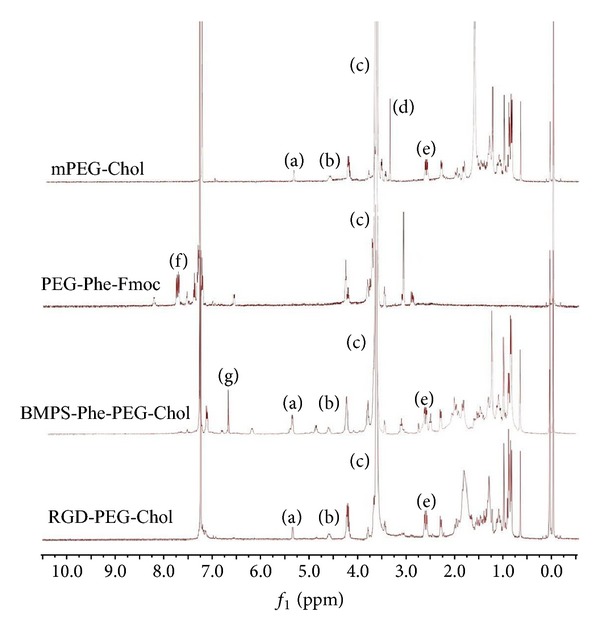
^1^H-NMR spectra of mPEG-PEG, PEG-Phe-Fmoc, BMPS-Phe-PEG-Chol, and RGD-PEG-Chol in CDCl_3_. (a, b) 6- and 3-position protons in cholesterol; (c) the repeating units of methoxyl in mPEG or PEG; (d) the protons in the terminal group of mPEG; (e) methylene proton of succinyl group; (f) protons in the Fmoc group; (g) protons on maleimidyl group of linkers.

As shown in Figure 1, the multiplet at *δ* 3.5–3.8 was attributed to the repeating units (–CH_2_, c) in PEG or mPEG. And the multiple signals at *δ* 7.40–7.78 came from benzene ring from Fmoc. The multiple signals at *δ* 0.67–2.20 came from the protons in cholesterol. The six-position protons (–CH=CH–, a) in cholesterol were found at *δ* 5.36. Signals at *δ* 7.12–7.36 were assigned to phenylalanine. As shown in the spectra of Linker-PEG-Chol, cholesterol and 3-maleimidopropionic acid linker had been connected to PEG chain successfully, and the peaks at *δ* 6.69 were attributed to the protons of linker (–CH=CH–, h). This result demonstrated that the linker had been conjugated to the Chol-PEG. The weak peaks at *δ* 4.0–4.2 belonged to protons of RGD (–NHCH(CH_2_)CO–, e) while peaks at *δ* 6.69 disappeared which indicated that the –CH=CH– had been broken. The whole results told us that the synthesis of RGD-PEG-Chol was successful.

**Figure 2:**
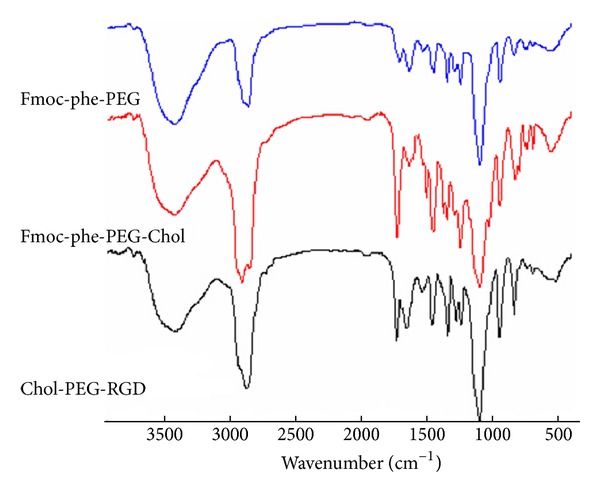
The FT-IR spectra of Fmoc-phe-PEG, Fmoc-phe-PEG-Chol, and RGD-PEG-Chol.

The FT-IR spectra of Fmoc-phe-PEG, Fmoc-phe-PEG-Chol, and Chol-PEG -RGD were shown in Figure 2. The broad band at around 3440 cm^−1^ was attributed to the –NH_2_ stretching vibration of phenylalanine. The sharp peak at 1733 cm^−1^ was assigned to the –C=O of –COOCO– group which was brought by the cholesterol monosuccinate. The peak at 2880 cm^−1^ was assigned to the –CH_2_CH_2_O– which was brought by PEG. Other prominent peaks at 1282 cm^−1^ and 1246 cm^−1^ were assigned to the asymmetrical and symmetrical bending vibrations of –C–S– which were the introduction of RGD chain after combination of –SH from cysteine to the linker.

### 3.2. Screening of Blank Liposome

**Figure 3:**
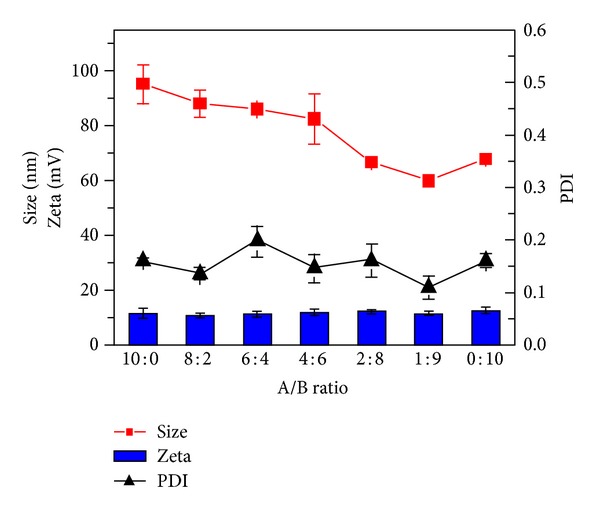
Screening of blank liposome.

The base lipid used in this experiment is cholesterol 13 mg, soy lecithin 39 mg, and the added polymers A: mPEG-Chol and B: RGD-PEG-Chol. The weight of A plus B is kept at 10 mg, and different A/B mass ratios were tested in the preparation process of liposomes. The results (Figure 3) show that as B RGD-PEG-Chol mass ratio increases, the particle size decreased from about 100 nm to 65 nm and that when mass ratios of A/B = 2 : 8 and 1 : 9, it achieved a smaller particle size, but when there was no polymer A, the particle size increased again. PDI and zeta potential of liposomes showed no obvious trend when the mass ratio of A/B changes from 10 : 0 to 0 : 10.

### 3.3. The Drug Loading and Final Screening of A/B Mass Ratio

Based on the results of previous section, we selected A/B mass ratios of 2/8 and 1/9, which bring optimum particle size and PDI results. Paclitaxel was employed in liposome formulation. In this part, we set different mass ratios of (A + B)/PTX as 10 : 1, 15 : 1, 20 : 1, and 30 : 1 to prepare liposomes encapsulating various amount of PTX. Then their size, PDI, zeta potential, drug loading (DL%), and the encapsulation efficiency (EE%) were determined. It is confirmed (Tables 1 and 2) that, when A/B mass ratio was set as 1 : 9, the parameters were better than those with A/B mass ratio of 2 : 8, and when we set mass ratio of (A + B)/PTX as 15 : 1, after comprehensive assessment of particle size, PDI, EE%, and DL%, we got optimal PTX-liposome, which has a size of 58.94 nm, a PDI of 0.154, zeta potential of 16.3 mv, DL% of 1.15%, and EE% of 99.12%.

### 3.4. Characterization of RGD-Modified PTX-Loaded Liposome

**Figure 4:**
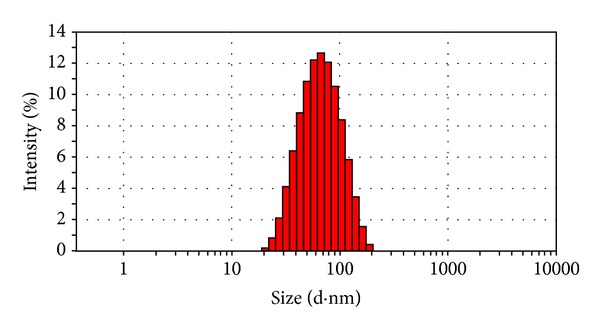
Size distribution of RGD-PTX liposomes.

**Figure 5:**
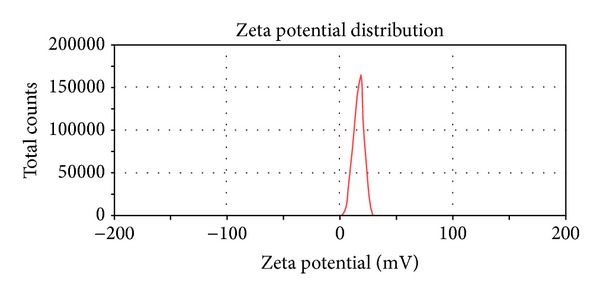
Zeta of RGD-PTX liposomes.

**Figure 6:**
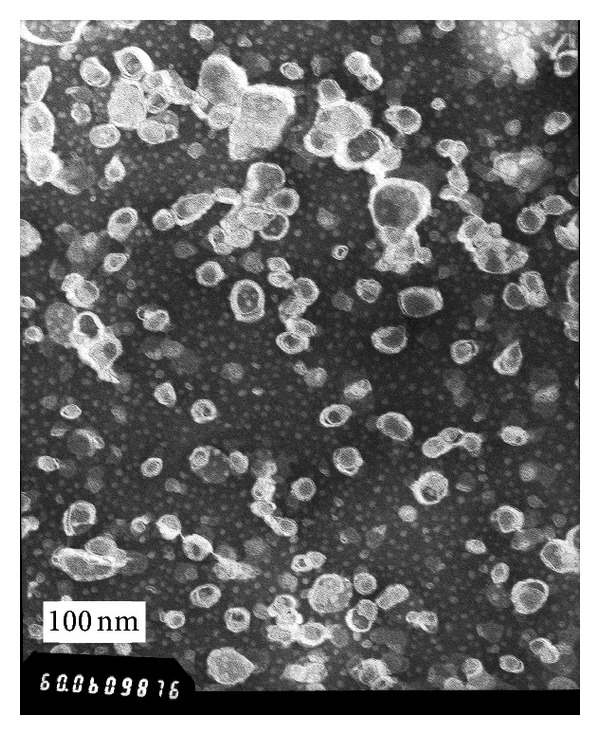
Typical TEM images of RGD-PTX liposomes.

The mean size of RGD-modified PTX-loaded liposome (RGD-PTX liposome) was 58.94 ± 4.82 (mean ± SD; *n* = 3) as shown in Figure 4, polydispersity index of 0.154 ± 0.10 (mean ± SD; *n* = 3), with zeta potential of 16.3 ± 0.1 mv (mean ± SD; *n* = 3) which was shown in Figure 5. When those preparations were stored at 4°C, there is no significant change of size within 28 days (data not shown). Further transmit electronic microscopy analyses confirmed that the liposomes were kept in a good form with suitable size which was shown in Figure 6. The HPLC results showed that the load dose of RGD-PTX liposome was >1% and the entrapment efficiency was >99%.

Drug-containing liposomes which have diameters with ranges of approximately 60–150 nm are small enough to extravasate from the blood vessels into the tumor interstitial space through their pores. In contrast, the blood vessels in most normal tissues are nonfenestrated capillaries and liposome with ~120 nm in mean diameter did not accumulate in these normal tissues after *i.v. *injection. The particle size of RGD-PTX liposome was below 100 nm with narrow distribution and high entrapment efficiency [31]. And this encourages us to hypothesize it may be possible that the liposome we made could obtain an improved therapeutic efficacy since it may accumulate more readily in tumor tissues.

### 3.5. Stability Test for RGD/PTX Liposomes

**Figure 7:**
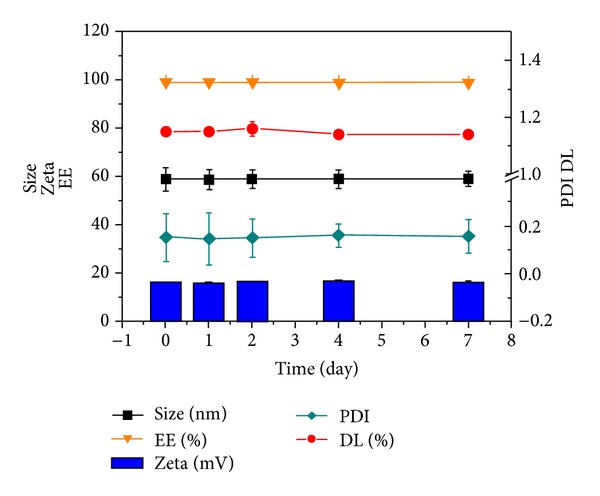
The stability of RGD/PTX liposomes in 7 days.

Liposomes were sealed at 4°C in refrigerator, and the associated data were measured at the set time point; the results confirmed that the prepared RGD/PTX liposomes were stable and showed no significant change in seven days (shown in Figure 7).

### 3.6. *In Vitro* Release Behavior of RGD/PTX Liposome

**Figure 8:**
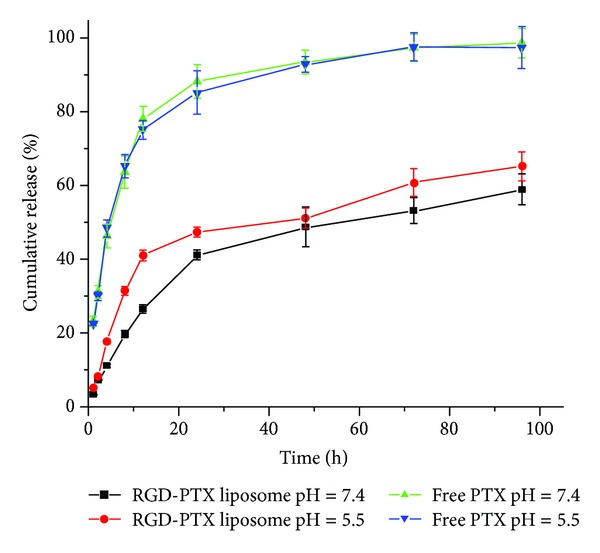
Drug release profiles of free PTX and RGD-PTX liposome in PBS solution at pH 7.4 and pH 5.8 with error bars showing the standard deviation (*n* = 3).

Figure 8 shows the *in vitro* release profiles of free PTX and PTX-loaded RGD liposome in PBS at pH 5.5 and at pH 7.4. As expected, in PBS at pH 5.5 and pH 7.4 free PTX showed a very fast release profile, whereas RGD-PTX liposome released PTX in a much slower way in both release media. In the first 24 hours, approximately up to 90% of drug was released in free PTX. In comparison, within the same period only 40% of PTX in RGD liposome in PBS at PH 7.4 was released, and less than 50% of PTX was released in PBS at pH 5.5. After 24 hours the release rate of PTX slowed down compared with the previous period of time and there was clearly a sustained release for at least 96 hours. The cumulative release rate of RGD-PTX liposome was 59.02 ± 4.09 at pH 7.4 and 65.32 ± 3.88 at pH 5.5, much slower than that of free PTX (98.67 ± 4.02). RGD-PTX liposome released PTX slightly faster in PBS with lower pH which indicated that RGD-PTX liposome might be pH-sensitive, which can be explained by faster hydrolysis of lipids at lower pH. Also, this phenomenon can be attributed to the tendency that the oxygen atom on the hydrophilic end of RGD-PEG-Chol polymer would absorb more positive hydrogen ions and produce electrostatic repulsion which would destabilize liposome and increase drug release rate [32]. Because the microenvironments of intratumoral regions and intracellular compartments such as endosomes and lysosomes are acidic, RGD-PTX liposome might selectively deliver and release PTX to tumor tissue, indicating their potential applicability as a drug delivery system with reduced side effects to healthy tissues and increased drug concentration at the tumor site.

### 3.7. Cellular Uptake of Liposomes by Flow Cytometry Analysis

**Figure 9:**
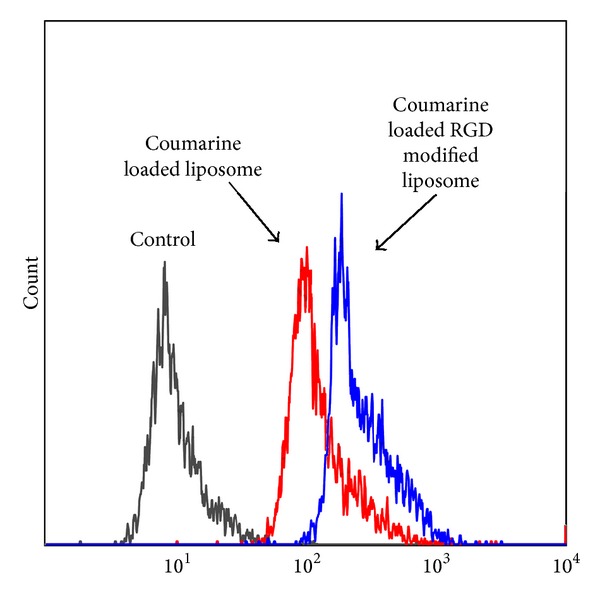
Flow cytometry profiles showed different cellular uptake of coumarin loaded liposomes with or without Chol-PEG-Chol by HUVEC cells (containing 40 ng/mL coumarin at 37°C for 1 hour).

A flow cytometry analysis was used to determine the cellular uptake of mPEG/coumarin-6 liposome, RGD/coumarin-6 liposome, blank liposome (control).  Figure 9 showed that uptake of RGD/coumarin-6 liposome by HUVECs was more than that of mPEG/coumarin-6 liposome after 1 h incubation. The mean fluorescence intensity of coumarin-6 uptaken by HUVECs treated with RGD liposome was increased by 50 percent (mean = 15.87 versus 23.53 arbitrary units) compared with that treated with mPEG liposome. These results confirmed that RGD modification enhanced uptake of liposomal drug by integrin-overexpressing HUVECs.

### 3.8. Cell Cytotoxicity of RGD-PTX Liposome

**Figure 10:**
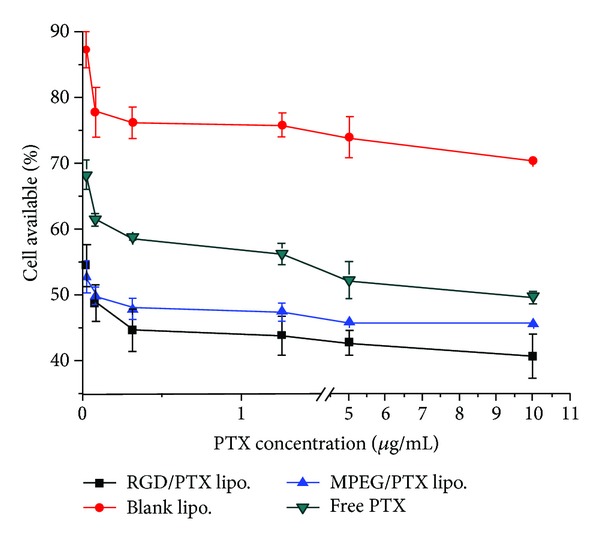
Cytotoxicity of free PTX, blank liposome, PTX liposome, and RGD-PTX liposome for B16F10 tumor cells.

The cytotoxicity of RGD-PTX liposome, PTX liposome, blank liposome, and free PTX to B16F10 cells was compared. As shown in Figure 10, the IC_50_ value of RGD-PTX liposome was decreased to 0.079 *μ*g/mL compared with 9.57 *μ*g/mL of free PTX and 0.302 *μ*g/mL of PTX liposome, respectively. The RGD-PTX liposome revealed more than 121-fold and 4-fold increase of cytotoxicity in comparison to free PTX and PTX liposome. The blank liposomes show no apparent cytotoxicity to B16F10 cells. The results may be explained by two mechanisms: firstly, the RGD fragments chemically conjugated to the Chol-PEG polymer enabled the PTX-loaded liposome to effectively target the surface of B16F10, since increased expression of *α*_v_ *β*_3_ integrins was seen in melanoma cells and the RGD peptide can specifically bind to the *α*_v_ *β*_3_ integrins on melanoma tumor cells [33]; secondly, the RGD-PTX liposome drug delivery system increased solubility of poorly soluble drugs, which persistently released the drug and enhanced the effect of PTX.

## 4. Conclusions 

In summary, a novel amphiphilic polymer Chol-PEG-RDG has been synthesized and characterized. The RGD/PTX liposomes have been successfully developed as a potential drug delivery system and the poorly soluble antitumor drug paclitaxel as model is loaded into the liposome. The liposome is spheroid with regular shape, a size within 100 nm and showed increased cellular uptake and sustained *in vitro *drug release behavior. Flow cytometry analysis revealed that the cellular uptake of coumarin encapsulated in the RGD-PEG-Chol modified liposome was increased for HUVEC cells. The* in vitro *cytotoxicity evaluated on the B16F10 cell lines by the MTT assay shows that RGD-PTX liposome has significantly enhanced cytotoxicity of paclitaxel for melanoma cells. Although further investigation on the amphiphilic polymer is required, the findings of our study represent an important step in advancing the use of RGD-PEG-Chol polymer modified liposome as a potential strategy to novel drug delivery system.

## Figures and Tables

**Table 1:** Data of PTX liposome when mass ratio of A/B is 1 : 9.

(A + B)/PTX	Size/nm	PDI	Zeta/mv	DL/%	EE%
10 : 1	110.3 ± 5.15	0.198 ± 0.31	20.1 ± 0.3	1.62 ± 0.021	97.25 ± 0.13
15 : 1	58.94 ± 4.82	0.154 ± 0.10	16.3 ± 0.1	1.15 ± 0.016	99.12 ± 0.07
20 : 1	62.49 ± 3.77	0.130 ± 0.21	14.7 ± 0.5	0.08 ± 0.008	99.56 ± 0.02
30 : 1	61.18 ± 2.15	0.113 ± 0.17	14.4 ± 0.7	0.05 ± 0.005	99.79 ± 0.02

Note: A: mPEG-Chol B: RGD-PEG-Chol A/B = 1 : 9.

**Table 2:** Data of PTX liposome when mass ratio of A/B is 2 : 8.

(A + B)/PTX	Size/nm	PDI	Zeta/mV	DL/%	EE%
10 : 1	111.9 ± 7.8	0.175 ± 0.5	26.5 ± 0.3	1.48 ± 0.017	95.11 ± 0.24
15 : 1	71.36 ± 6.1	0.192 ± 0.17	19.1 ± 0.7	1.06 ± 0.022	98.85 ± 0.33
20 : 1	63.76 ± 5.2	0.172 ± 0.32	16.7 ± 1.1	0.81 ± 0.015	99.12 ± 0.25
30 : 1	66.11 ± 4.7	0.144 ± 0.11	13.2 ± 0.8	0.53 ± 0.012	99.7 ± 0.19

Note: A: mPEG-Chol B: RGD-PEG-Chol A/B = 2 : 8.

## References

[1] McHugh AJ (2005). The role of polymer membrane formation in sustained release drug delivery systems. *Journal of Controlled Release*.

[2] Bajpai AK, Shukla SK, Bhanu S, Kankane S (2008). Responsive polymers in controlled drug delivery. *Progress in Polymer Science*.

[3] Sershen S, West J (2002). Implantable, polymeric systems for modulated drug delivery. *Advanced Drug Delivery Reviews*.

[4] Halliday AJ, Moulton SE, Wallace GG, Cook MJ Novel methods of antiepileptic drug delivery—polymer-based implants. *Advanced Drug Delivery Reviews*.

[5] Lukyanov AN, Torchilin VP (2004). Micelles from lipid derivatives of water-soluble polymers as delivery systems for poorly soluble drugs. *Advanced Drug Delivery Reviews*.

[6] Wang Y, Grayson SM (2012). Approaches for the preparation of non-linear amphiphilic polymers and their applications to drug delivery. *Advanced Drug Delivery Reviews*.

[7] Rostamizadeh K, Vahedpour M, Bozorgi S (2012). Synthesis, characterization and evaluation of computationally designed nanoparticles of molecular imprinted polymers as drug delivery systems. *International Journal of Pharmaceutics*.

[8] Yang D-B, Zhu J-B, Huang Z-J, Ren H-X, Zheng Z-J (2008). Synthesis and application of poly(ethylene glycol)-cholesterol (Chol-PEG^m^) conjugates in physicochemical characterization of nonionic surfactant vesicles. *Colloids and Surfaces B*.

[9] Carrion C, Domingo JC, De Madariaga MA (2001). Preparation of long-circulating immunoliposomes using PEG-cholesterol conjugates: effect of the spacer arm between PEG and cholesterol on liposomal characteristics. *Chemistry and Physics of Lipids*.

[10] Levchenko TS, Rammohan R, Lukyanov AN, Whiteman KR, Torchilin VP (2002). Liposome clearance in mice: the effect of a separate and combined presence of surface charge and polymer coating. *International Journal of Pharmaceutics*.

[11] Dadashzadeh S, Vali AM, Rezaie M (2008). The effect of PEG coating on *in vitro* cytotoxicity and *in vivo* disposition of topotecan loaded liposomes in rats. *International Journal of Pharmaceutics*.

[12] Lin G, Cosimbescu L, Karin NJ, Tarasevich BJ (2012). Injectable and thermosensitive PLGA-g-PEG hydrogels containing hydroxyapatite: preparation, characterization and in vitro release behavior. *Biomedical Materials*.

[13] Liu D, Wang T, Liu XX, Tong Z Accelerated cell sheet detachment by copolymerizing hydrophilic PEG side chains into PNIPAm nanocomposite hydrogels. *Biomedical Materials*.

[14] Garbuzenko O, Barenholz Y, Priev A (2005). Effect of grafted PEG on liposome size and on compressibility and packing of lipid bilayer. *Chemistry and Physics of Lipids*.

[15] Wang Y-Y, Lü L-X, Feng Z-Q, Xiao Z-D, Huang N-P (2010). Cellular compatibility of RGD-modified chitosan nanofibers with aligned or random orientation. *Biomedical Materials*.

[16] Bon RS, Waldmann H (2010). Bioactivity-guided navigation of chemical space. *Accounts of Chemical Research*.

[17] Hersel U, Dahmen C, Kessler H (2003). RGD modified polymers: biomaterials for stimulated cell adhesion and beyond. *Biomaterials*.

[18] Pastorino F, Brignole C, Di Paolo D (2006). Targeting liposomal chemotherapy via both tumor cell-specific and tumor vasculature-specific ligands potentiates therapeutic efficacy. *Cancer Research*.

[19] Ruoslahti E (1996). RGD and other recognition sequences for integrins. *Annual Review of Cell and Developmental Biology*.

[20] Spratlin J, Sawyer MB (2007). Pharmacogenetics of paclitaxel metabolism. *Critical Reviews in Oncology/Hematology*.

[21] Javeed A, Ashraf M, Riaz A, Ghafoor A, Afzal S, Mukhtar MM (2009). Paclitaxel and immune system. *European Journal of Pharmaceutical Sciences*.

[22] Thompson B, Mignet N, Hofland H (2005). Neutral postgrafted colloidal particles for gene delivery. *Bioconjugate Chemistry*.

[23] Leblond J, Mignet N, Leseurre L (2006). Design, synthesis, and evaluation of enhanced DNA binding new lipopolythioureas. *Bioconjugate Chemistry*.

[24] Zhao XB, Muthusamy N, Byrd JC, Lee RJ (2007). Cholesterol as a bilayer anchor for PEGylation and targeting ligand in folate-receptor-targeted liposomes. *Journal of Pharmaceutical Sciences*.

[25] Xu J-P, Ji J, Shen J-C (2009). The effect of a cholesterol liquid crystalline structure on osteoblast cell behavior. *Biomedical Materials*.

[26] Qin Y, Chen H, Yuan W (2011). Liposome formulated with TAT-modified cholesterol for enhancing the brain delivery. *International Journal of Pharmaceutics*.

[27] Coderch L, Fonollosa J, De Pera M, Estelrich J, De La Maza A, Parra JL (2000). Influence of cholesterol on liposome fluidity by EPR. Relationship with percutaneous absorption. *Journal of Controlled Release*.

[28] Qian X, Lai J, Zhan S, Zhang J, Wang L, Tu K Novel mePEG-ehol and mePEG-st mixed micellar systems for drug delivery: preparation and characterization. *Rare Metal Materials and Engineering*.

[29] Wu F, Liu T, Chen C, Song C, Zheng XR, He G (2013). Glycyrrhetinic acid-poly(ethylene glycol)-glycyrrhetinic acid tri-block conjugates based self-assembled micelles for hepatic targeted delivery of poorly water soluble drug. *The Scientific World Journal*.

[30] Mahmood T, Akhtar N (2013). Stability of a cosmetic multiple emulsion loaded with green tea extract. *The Scientific World Journal*.

[31] Xiong X-B, Huang Y, Lu W-L (2005). Enhanced intracellular delivery and improved antitumor efficacy of doxorubicin by sterically stabilized liposomes modified with a synthetic RGD mimetic. *Journal of Controlled Release*.

[32] Li JM, He ZY, Yu S (2012). Micelles based on methoxy poly (ethylene glycol)-cholesterol conjugate for controlled and targeted drug delivery of a poorly water soluble drug. *Journal of Biomedical Nanotechnology*.

[33] Li W, Su B, Meng S (2011). RGD-targeted paramagnetic liposomes for early detection of tumor: *in vitro* and *in vivo* studies. *European Journal of Radiology*.

